# Gellan-Based Composite System as a Potential Tool for the Treatment of Nervous Tissue Injuries: Cross-Linked Electrospun Nanofibers Embedded in a RC-33-Loaded Freeze-Dried Matrix

**DOI:** 10.3390/pharmaceutics13020164

**Published:** 2021-01-26

**Authors:** Barbara Vigani, Caterina Valentino, Valeria Cavalloro, Laura Catenacci, Milena Sorrenti, Giuseppina Sandri, Maria Cristina Bonferoni, Chiara Bozzi, Simona Collina, Silvia Rossi, Franca Ferrari

**Affiliations:** Department of Drug Sciences, University of Pavia, Viale Taramelli, 12, 27100 Pavia, Italy; barbara.vigani@unipv.it (B.V.); caterina.valentino01@universitadipavia.it (C.V.); valeria.cavalloro01@universitadipavia.it (V.C.); laura.catenacci@unipv.it (L.C.); milena.sorrenti@unipv.it (M.S.); giuseppina.sandri@unipv.it (G.S.); cbonferoni@unipv.it (M.C.B.); chiara.bozzi01@universitadipavia.it (C.B.); simona.collina@unipv.it (S.C.); franca.ferrari@unipv.it (F.F.)

**Keywords:** nervous tissue injuries, gellan gum, S1R agonist, electrospinning, nanofibers, cross-linking, RC-33/GG interaction product, freeze-drying, porous matrices

## Abstract

Injuries to the nervous system affect more than one billion people worldwide, and dramatically impact on the patient’s quality of life. The present work aimed to design and develop a gellan gum (GG)-based composite system for the local delivery of the neuroprotective sigma-1 receptor agonist, 1-[3-(1,1′-biphen)-4-yl] butylpiperidine (RC-33), as a potential tool for the treatment of tissue nervous injuries. The system, consisting of cross-linked electrospun nanofibers embedded in a RC-33-loaded freeze-dried matrix, was designed to bridge the lesion gap, control drug delivery and enhance axonal regrowth. The gradual matrix degradation should ensure the progressive interaction between the inner fibrous mat and the surrounding cellular environment. Nanofibers, prepared by electrospinning polymeric solutions containing GG, two different grades of poly (ethylene oxide) and poloxamer, were cross-linked with calcium ions. GG-based matrices, loaded with different amounts of RC-33, were prepared by freeze-drying. Dialysis studies and solid-state characterization pointed out the formation of an interaction product between GG and RC-33. RC-33-loaded freeze-dried matrices were characterized by the capability to absorb a high buffer content, forming a gel with marked viscoelastic properties, and by RC-33 controlled release properties. The presence of cross-linked nanofibers increased matrix mechanical resistance.

## 1. Introduction

Injuries to the nervous system affect more than one billion people worldwide, and dramatically impact on the patient’s quality of life [1,2,3]. Such lesions, generally caused by mechanical, ischemic and/or chemical events, are characterized by the loss of neurons and/or neuroglial cells, which leads to the formation of a cavitary defect. This tissue discontinuity results in a disruption of the neural network and, thus, in an impairment of some fundamental system functions, like memory, cognition, language and voluntary movement [3]. Particularly devastating neurological disabilities, such as loss of sensory/motor capabilities and paralysis under the level of damage, may result from spinal cord injury (SCI) [4,5,6].

Nowadays, the restoration of the injured nervous tissue still remains challenging and therapeutic approaches ensuring full functional recovery are often ineffective. In this scenario, neural tissue engineering aims to design and produce biomaterial-based scaffolds that fill the gap at the injury site, providing a biodegradable structure that support and guide axonal outgrowth, thus repairing the broken neural circuitry [7,8,9,10,11]. According to biomimetic principles, an ideal neural scaffold should be characterized by a porous interconnected structure that resembles that of the native extracellular matrix (ECM) and, simultaneously, by well-balanced mechanical properties, which allow the scaffold to support cell adhesion and migration, without stressing excessively the injured area or collapsing throughout regular motion [12,13,14].

Among biomimetic scaffolds, polymeric fibers have proved to be effective therapeutic platforms at supporting the regeneration of damaged nervous tissues, particularly due to their large surface area-to-volume ratio that maximizes the contact with the surrounding cells at the injury site. Electrospinning has been recognized as one of the most compelling technique used for the production of micro- and nanofibers with tunable morphological properties; in the case of nervous tissue injuries, the optimal fiber size ranges from 200 nm to a few micrometers, depending on the damage localization [7,8,11].

Freeze-drying is another technique widely used in the production of neural scaffolds based on natural materials, such as polysaccharides. After application at the injury site, freeze-dried matrices, characterized by an interconnected structure, absorb the biological fluids, such as the cerebrospinal one, gradually turning into gels, which resemble the hydrated ECM microenvironment in which cells could migrate [7,11].

In recent years, gellan gum (GG) has become an attractive biomaterial for regenerative medicine purposes due to its hydrogel-forming properties when combined with some monovalent (Na^+^) and/or divalent (Mg^2+^ and Ca^2+^) cations present in physiological fluids [15,16,17]. GG is an exocellular polysaccharide produced by *Sphingomonas elodea* bacteria, characterized by a linear anionic structure composed of repeated tetrasaccharide units (1,3-β-d-glucose, 1,4-β-d-glucuronic acid, 1,4-β-d-glucose and 1,4-α-l-rhamnose). In neural applications, GG-based hydrogels have been used as luminal fillers of tubular scaffolds [18,19] and/or as vehicles for the transplantation of neural stem cells [20,21,22] and olfactory ensheathing glia cells [20,23,24].

However, a biomaterial-based neural scaffold is not always sufficient for the repair of nervous tissue lesions, especially in the central nervous system (CNS), where the injury site generally corresponds to an inhibitory environment that impedes any regenerative strategies [3]. In such cases, neuroprotective drug-based therapies are fundamental to prevent the spread of the secondary injury and facilitate regeneration [12]. In the last decade, sigma 1 receptors (S1R) have gained attention as new putative therapeutic targets for their involvement in the progression of several post-injury neurodegenerative processes, such as glutamate excitotoxicity and reactive oxygen species (ROS) production, affecting both neurons and glial cells in the nervous system [25,26,27,28,29,30]. Over the years, Rossi and colleagues designed and synthetized a S1R modulator compound library, based on arylalkyl (alkenyl) aminic scaffold, leading to the identification of 1-[3-(1,1′-biphen)-4-yl]butylpiperidine (RC-33), as a molecule characterized by high receptor affinity, favorable physicochemical properties and promising pharmacological efficacy [31,32,33,34]. In particular, in vitro studies performed on PC12 cell line (derived from a rat adrenal pheochromocytoma) have demonstrated that RC-33 was able to potentiate neurite outgrowth (from 0.01 to 5 μM) and elongation (from 0.25 to 5 μM) [35].

In a previous work of ours, a dual-functioning scaffold for the local delivery of RC-33 was proposed as a combined approach, ensuring both neuroprotective and neuroregenerative outcomes, in the treatment of SCI [36]. In particular, RC-33-loaded nanofibers, based on alginate (ALG) and prepared by electrospinning, were incorporated in chitosan films to obtain a drug delivery system endowed with high flexibility, that was functional to an easy application at the injury site, and characterized by a controlled biodegradation rate.

An improvement of the above-mentioned approach may be represented by the design of a composite system consisting of polymeric nanofibers (inner component) enclosed in a porous solid matrix loaded with the neuroprotective drug candidate (outer component). In particular, the matrix should be characterized by a structure mimicking the ECM architectural template, ease to handle and shapeable according to the lesion size and morphology. Once applied at the site of injury, the porous structure of the system should allow the absorption and the drainage of biological fluids, thus reducing the intrathecal pressures in the case of SCI; the resulting hydrated system should be responsible for a controlled drug release. Finally, the gradual matrix degradation should ensure the progressive interaction between the inner fibrous mat and the surrounding cellular environment.

In this perspective, the present work deals with the formulation development of a GG-based composite system, consisting of cross-linked electrospun nanofibers (cNFs; inner structure) embedded in a RC-33-loaded freeze-dried matrix (RC-33/MX; outer structure). GG was selected as the principal polymer of the composite system especially due to its anionic nature: the formation of an interaction product (IP) between GG and the cationic RC-33 should be responsible for a controlled drug release.

In detail, the inner cNFs were prepared by cross-linking electrospun nanofibers (NFs) composed of GG, two grades of poly (ethylene oxide) (PEO) and poloxamer (P407). cNFs were characterized for size and morphology. The outer RC-33/MXs were prepared by freeze-drying polymeric solutions (RC-33/SLs), based on GG and containing different RC-33 concentrations, chosen on the basis of their rheological properties. A particular attention has been devoted to the investigation (stoichiometry and solid-state characterization) of the GG/RC-33 IP forming upon mixing RC-33 and GG aqueous solutions. RC-33/MXs were characterized for porosity, mechanical/rheological properties before and after hydration and capability to prolong RC-33 release. Moreover, the influence of CaCl_2_ incorporation into RC-33/MXs on their mechanical/rheological and release properties was investigated.

Finally, the composite system, consisting of cNFs embedded in the most promising RC-33/MX, was prepared and characterized for mechanical properties.

## 2. Materials and Methods

### 2.1. Materials

For the preparation of polymeric solutions the following materials were used: Deacetylated gellan gum (GG, Gelrite^®^, Kelco Division of Merck & Co., Rahway, NJ, USA), Kolliphor P407 poloxamer (P407, BASF SE, Ludwigshafen, Germany), poly (ethylene oxide) of high molecular weight (h-PEO, MW = 4000 kDa, Colorcon, Dartford, UK), poly (ethylene oxide) of low molecular weight (l-PEO, MW = 600 kDa, Sigma Aldrich, Milan, Italy) and glycine (Gly, Sigma Aldrich, Milan, Italy). GG-based NFs cross-linking was performed using anhydrous calcium chloride (CaCl_2_) and absolute ethanol (EtOH; Carlo Erba Reagents S.r.l., Milan, Italy).

Reagents and solvents for RC-33 synthesis were purchased from Merck (Milan, Italy).

### 2.2. Cross-Linked Electrospun Nanofibers (cNFs)

#### 2.2.1. NFs Preparation and Characterization

Four polymeric solutions (G1–G4), containing 1.5% *w*/*w* GG, l-PEO and h-PEO were prepared in distilled water (Table 1); P407 was added at the concentration of 2% *w*/*w* in order to reduce the surface tension of the GG/PEO blends. The polymeric solutions were maintained under stirring overnight at room temperature before electrospinning.

All the polymeric solutions, after characterization in terms of rheological and electrical properties, were electrospun by using an electrospinning apparatus (STIKIT-40; Linari Engineering, Grosseto, Italy), equipped with a high-voltage power supply (Linari Engineering), a syringe pump (Razel Scientific, Saint Albans, VT, USA) and a collector plate (Linari Engineering), covered by an aluminum foil. Spinneret-collector distance, applied voltage and flow rate were investigated in order to identify the best electrospinning conditions for each formulation. The best electrospinning conditions were 20 cm distance and 20 kV voltage for all the samples considered. Polymeric solutions were pumped through a needle with a length = 15 mm and a gauge = 21; the electrospinning process was performed at atmospheric pressure, maintaining constant temperature and relative humidity ranges, 27 °C–33 °C and 20%–30%, respectively.

Nanofibers morphology was investigated by means of a scanning electron microscope (Tescan Mira3 XMU, Brno, Czech Republic). NFs were carefully handled: They were detached from the aluminum foil and placed with a tweezer on the microscope stub.

NFs size was measured using the imaging analysis program ImageJ 2.0 (net.imagej:imagej:2.0.0-rc-55, Java-based operating system, 2009); thirty fibers were considered for each sample. NFs thickness was measured by means of a Sicutool 3955G-50 (Milan, Italy) apparatus.

#### 2.2.2. NFs Cross-Linking

NFs cross-linking was carried out according to a multistep protocol, slightly modified as described by [37]. Briefly, NFs were subjected to a pre-cross-linking treatment with absolute ethanol; subsequently, NFs were soaked in 2% *w*/*w* CaCl_2_ ethanol/water (80:20 *w*/*w*) solution for 15 min and, then, in 3% *w*/*w* CaCl_2_ ethanol/water (50:50 *w*/*w*) solution for the same time. After 1 h of soaking in 5% *w*/*w* CaCl_2_ aqueous solution, cNFs were, finally, washed with distilled water and dried at room temperature. Morphological analysis on cNFs was also performed (as described in the Section 2.2.1.).

### 2.3. RC-33/GG Interaction Product (RC-33/GG IP)

#### 2.3.1. RC-33/GG Binding Study by Dialysis Equilibrium

RC-33 was synthetized and characterized, as described in [32,36]. Dialysis equilibrium studies were performed to investigate the polymer/drug interaction, in particular to quantify the amount of RC-33 bound to 1 mg of GG, as described by [36]. Briefly, a dialysis bag (seamless cellulose tubing, cut-off > 12,000 Da; Sigma Aldrich, Milan, Italy) containing 1.5% *w*/*v* GG solution was dialyzed for 24 h towards a RC-33 HCl solution under mild stirring at room temperature. Different RC-33 HCl concentrations were considered: 1, 3, 10 and 15 mg/mL.

After 24 h, drug concentration outside the dialysis bags was quantified by a HPLC method as described in [36].

#### 2.3.2. Solid-State Characterization

GG/RC-33 IP, formed in the dialysis bag in presence of 10 mg/mL RC-33, was dried at 50 °C for three days and subjected to a solid-state characterization in comparison with GG and RC-33.

Differential scanning calorimetry (DSC) analysis (10–300 °C, heating rate β = 10 K min^−1^, nitrogen air atmosphere (flux 50 mL min^−1^)) was performed by means of Mettler STAR^e^ thermal analysis system, version 9.30 DSC821^e^ (Mettler Toledo, Milan, Italy), equipped with an Intracooler device for sub-ambient temperature analysis (Julabo FT 900). Three replicates were performed for each sample.

Thermogravimetric analysis (TGA) (30–300 °C, heating rate β = 10 K min^−1^, nitrogen air atmosphere (flux 50 mL min^−1^), temperature range) was carried out by means of a Mettler STAR^e^ system (Mettler Toledo, Milan, Italy). Three replicates were performed for each sample.

Fourier-transform infrared spectroscopy (FT-IR) spectra were recorded using a FT-IR spectrophotometer (Perkin Elmer Spectrum One, Monza, Italy) with a single reflection attenuated total reflectance (ATR) accessory (PIKE MIRacle™). Approximately 5 mg of each sample (RC-33, GG and RC-33/GG IP) were placed on ATR crystal of ZnSe and pressed down to the crystal. The scanning range was 650–4000 cm^−1^ and the resolution was 4 cm^−1^.

### 2.4. Freeze-Dried Matrices (MXs)

RC-33/MXs and blank/MX were prepared by freeze-drying 1.5% *w*/*w* GG solutions, in presence (RC-33/SLs) and in absence (blank/solution (SL)) of RC-33, respectively, as hereafter described. In all GG SLs, Gly was used as cryoprotectant agent at the concentration of 0.5% *w*/*w*.

#### 2.4.1. SL Preparation and Rheological Characterization

After the maximum binding capacity of GG for RC-33 was identified, four RC-33-loaded solutions (RC-33/SLs), containing 1.5% *w*/*w* GG and 0.5% *w*/*w* Gly, were prepared in distilled water by addition of RC-33 at four different concentrations. Such concentrations were calculated so that RC-33 could occupy 10% (10%RC-33/SL), 20% (20%RC-33/SL), 30% (30%RC-33/SL) and 40% (40%RC-33/SL) of GG binding sites in a solution containing GG at 1.5% *w*/*w*. A solution containing 1.5% *w*/*w* GG and 0.5% *w*/*w* Gly (blank/SL), without RC-33, was also prepared.

All SLs were subjected to rheological analysis. Viscosity measurements were carried out at 25 °C by means of a rotational rheometer (MCR 102, Anton Paar, Turin, Italy) equipped with a cone plate combination (CP50-1, diameter = 50 mm; angle = 1°) as measuring system. SL viscosity was investigated at increasing shear rates in the range 1–300 s^−1^. Three replicates were affected for each measurement.

#### 2.4.2. Blank/MX and RC-33/MXs Preparation

Blank/SL and RC-33/SLs were poured into the wells of a 12-well plate (2 g polymeric solution/well). SLs were frozen at −80 °C for 24 h and then subjected to sublimation (Heto Drier, Analitica De Mori, Milan, Italy) for 48 h.

#### 2.4.3. Blank/MX and RC-33/ MXs Characterization

##### Morphological and Porosity Properties

Morphological analysis was performed by means of a scanning electron microscope (Tescan Mira3 XMU, Brno, Czech Republic). MXs porosity was calculated as described in [38].

##### Mechanical Properties

Tensile test was performed by means of a TA.XT plus Texture Analyzer (Stable Micro Systems, Godalming, UK), equipped with a 5 kg load cell and a P/10 measuring system, consisting of a cylindrical probe with a diameter of 10 mm. Before testing, each MX was placed on the base of the instrument. The probe was lowered with a pre-test speed equal to 1.00 mm/s for a distance of 2.5 mm in order to determine deformation of the sample; afterwards the probe was raised with a post-test speed of 5.00 mm/s. The force required for MX compression was measured at three different distances: 1, 2 and 2.5 mm. Three replicates were carried out for each MX.

##### Hydration Properties

Hydration measurements were performed at 37 °C by means of Franz diffusion cells (PermeGear, Bethlehem, Palestine). In particular, a weighted portion of each MX was layered on a dialysis membrane in the apical chamber of the Franz cell. The receptor chamber was filled with phosphate buffer saline (PBS, pH 7.4), mimicking biological fluids and prepared according to European Pharmacopoeia 7.0. After 6 h, the sample weight was measured and the PBS absorption was calculated as Hydration Ratio % according to the following equation:

Hydration Ratio % = [(Wf − Wi)/Wi] × 100(1)

where Wf was the weight of the hydrated sample, while Wi was the weight of the dried one. For each sample, three replicates were performed.

##### Viscous and Elastic Properties after Hydration

After 6 h hydration, dynamic oscillatory measurements were performed by means of a rotational rheometer (MCR102, Anton Paar, Turin, Italy), using a parallel plate combination (PP25, diameter = 25 mm) as measuring system. In the oscillation test, a shear stress equal to 1 Pa was applied at increasing frequencies (1 to 20 Hz) and G’ (storage modulus) and G” (loss modulus) profiles were recorded; measurements were performed at 37 °C. Three replicates were considered for each sample.

After 6 h hydration, a tensile test was performed as previously described in the above paragraph “Mechanical Properties”. Young’s modulus (E) was calculated according to the following equation:

E = (F/A)/(Δl/l_0_) = σ/ε (2)

where σ was the tensile strength (F/A; compressive force per unit area) and ε was the axial strain (Δl/l_0_; proportional deformation, where Δl was the distance of compression and l_0_ was the MX thickness).

##### In Vitro Release Properties

Blank/MX and RC-33/MXs were enclosed in a dialysis bag (seamless cellulose tubing, cut-off > 12,000 Da; Sigma Aldrich, Milan, Italy), previously soaked at 80 °C for 15 min in distilled water and carefully washed. Each dialysis bag, placed in Petri dish DUROPLAN^®^ (60 by 20 mm; Sigma Aldrich, Milan, Italy) and dialyzed towards 15 mL of saline, was maintained in a heating/shaking water bath (FALC Instruments, Treviglio, Italy) at 37 °C and 60 rpm, for 48 h.

At fixed time end-points (30 min, 1 h, 2 h, 3 h, 6 h, 24 h and 48 h), 2 mL of saline was collected and replaced by the same volume of fresh saline, maintained at 37 °C. RC-33 concentration outside the dialysis bags was quantified by a HPLC method as described in the Section 2.3.1. Nine replicates were considered for each sample.

### 2.5. RC-33/MX Containing CaCl_2_ (40%RC-33/MX + CaCl_2_)

Then, 40%RC-33/SL was added with CaCl_2_ at the concentration of 0.035% *w*/*w*. CaCl_2_ concentration was calculated so that Ca^2+^ could occupy 30% of the remaining GG binding sites. Afterwards, the solution was freeze-dried as described in the Section 2.4.2. The 40%RC-33/MX + CaCl_2_ was characterized as described in the Section 2.4.3.

### 2.6. Composite System (cNFs + 40%RC-33/MX)

#### Preparation and Characterization

After cross-linking process (described in the Section 2.2.2.), cNFs were soaked in 40%RC-33/SL, when just poured into a Petri dish (50 × 20 mm). The composite system was frozen at −80 °C for 24 h and, then, subjected to sublimation (Heto Drier, Analitica De Mori, Milan, Italy) for 48 h. Composite system morphology and thickness were investigated as described in the Section 2.2.1.

The mechanical properties of the composite system (cNFs + 40%RC-33/MX) were assessed in comparison with 40%RC-33/MX by means of a TA.XT plus Texture Analyzer (Stable Micro Systems, Godalming, UK), equipped with 5 kg load cells.

Each sample was cut (1 by 3 cm) and then clamped on an A/TG tensile grips probe; an initial distance of 1 cm between the grips was set. The upper grip was raised at a constant speed of 10 mm/s. Deformation force and distance at break were measured and the deformation work was calculated as the area under the force vs. displacement curve (AUC). Three replicates were considered for each sample.

### 2.7. Statistical Analysis

Whenever possible, experimental values of the various types of measurements were subjected to statistical analysis, carried out by means of the statistical package Statgraphics 5.0 (Statistical Graphics Corporation, Rockville, MD, USA). In particular, *t*-test or one-way ANOVA one way followed by post hoc Sheffé test were carried out.

## 3. Results and Discussion

### 3.1. Cross-Linked Electrospun Nanofibers (cNFs)

In the last two decades, electrospinning has been recognized as a simple, low-cost and versatile technique for the fabrication of submicron fibers from polymeric solutions or blends. It consists in an electrohydrodynamic process: the polymeric droplet, that forms at the end of the syringe needle, is subjected to an electric field leading to the generation of a continuous jet, which stretches and elongates to form solid fibers with a diameter from nano- to micro-scale. Size and morphology of the resulting fibers can be influenced by different parameters, among which those related to the starting polymeric solution, such as polymer concentration and molecular weight (MW), viscosity, surface tension and conductivity [39,40]. In a previous work of ours, a design of experiments (DoE) approach (full factorial design) was proposed as useful tool to investigate, on a statistical basis, the role of each polymeric solution parameter on the production of homogeneous ALG-containing fibers, regardless the non-ionic polymer used to enhance ALG electrospinnability [37].

In the present study, GG, another anionic polysaccharide, was selected for the production of electrospun nanofibers. As is the case for most of natural polysaccharides, the electrospinning of GG is challenging due to its intrinsic properties, such as the low sol-gel transition concentration and a non-typical solvation in water. In fact, it has been demonstrated that a 1% GG aqueous solution produced only deformed droplets and an unstable Taylor cone at the spinneret, without being able to guarantee a continuous polymeric jet and, thus, the formation of homogenous fibers. These results could be explained by GG anionic nature, shear thinning behavior at low-shear rates and inability to form in water a number of chain entanglements sufficient to sustain the integrity of the jet [41,42].

The strategy most widely used to overcome these limitations is blending GG with a second synthetic non-ionic polymer, i.e., PEO or poly(vinyl alcohol) (PVA) [42,43,44].

Recently, in the case of ALG solutions, we have demonstrated that the addition of a small amount of PEO at high MW (h-PEO) to PEO at low MW (l-PEO) was responsible for the production of homogenous nanofibers endowed with optimal mechanical properties [45]. P407 was, instead, used in order to reduce the surface tension of the polymeric solution, thus facilitating its ejection from the spinneret [37].

Based on our experience, in the present work, GG was blended with a mixture of two different grades of PEO and P407. The rheological and electrical properties of four different polymeric solutions (G1–G4), characterized by fixed GG (1.5% *w*/*w*) and P407 (2% *w*/*w*) concentrations and different l-PEO and h-PEO amounts, have been investigated (see Supplementary Data: Figure S1). GG and P407 concentrations were chosen on the basis of the results of a preliminary study (data not shown) in which different GG concentrations (ranging from 0.8% to 1.5% *w*/*w*) and P407 concentrations (ranging from 1% to 3% *w*/*w*) were considered.

**Figure 1:**
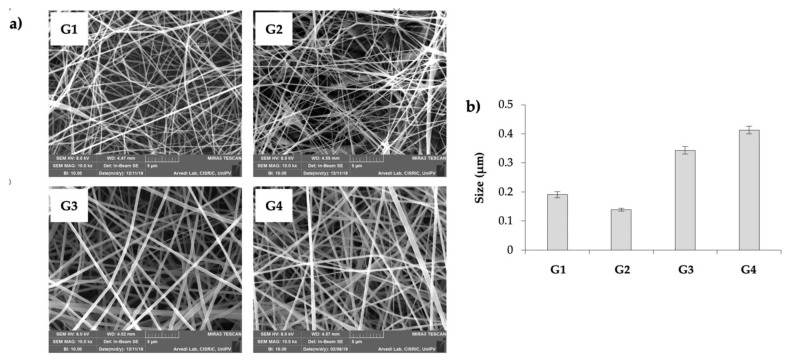
SEM micrographs (**a**) and size (mean values ± s.e.; *n* = 30) (**b**) of the electrospun nanofibers obtained from the different polymeric solutions (20 cm distance; 20 kV voltage). One-way ANOVA; post hoc Scheffé test (*p* ≤ 0.05): G1 vs. G3, G4; G2 vs. G3, G4; G3 vs. G4.

Figure 1 reports SEM micrographs (a) and size (b) of the electrospun nanofibers obtained from the above-mentioned polymeric solutions. Nanofibers are indicated with the same code used for the starting solutions. G3 and G4 fibers show the best morphology and the highest size homogeneity. G4 fibers are characterized by the largest size (Figure 1b), probably due to the highest h-PEO content. They are also characterized by the best electrospinnability and by the highest yield (after 1 h of electrospinning a fiber membrane with a thickness in a range of 50–70 μm is obtained), due to the continuity of the process that occurs without interruptions. For these reasons, G4 nanofibers were chosen for the prosecution of the work.

**Figure 2:**
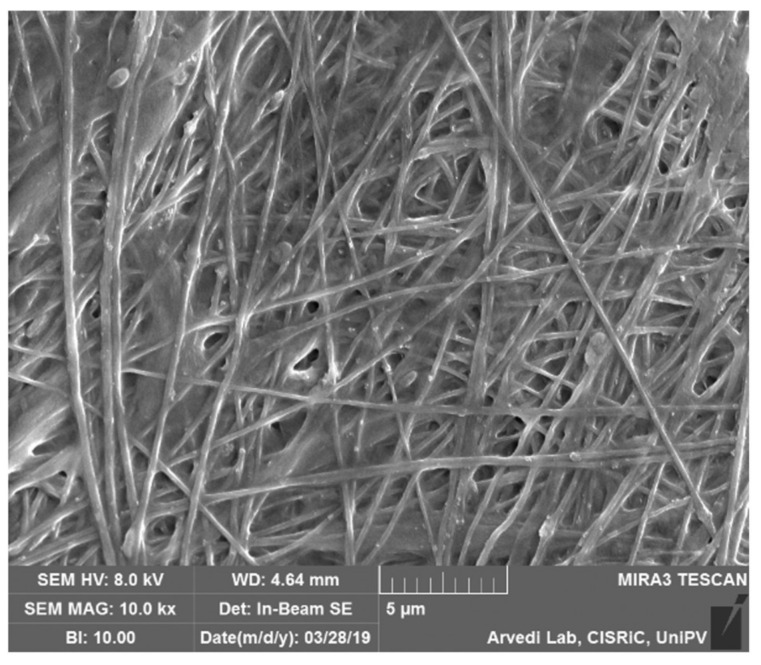
SEM micrograph of G4 nanofibers after cross-linking (cNFs).

In order to make G4 nanofibers insoluble in physiological fluids, they were subjected to a cross-linking process with CaCl_2_; Figure 2 reports SEM micrograph of the cross-linked nanofibers. The cross-linking process does not produce any significant variation in fiber size (one-way ANOVA, post hoc Scheffé test (*p* ≤ 0.05)), which are characterized by a mean diameter equal to 0.389 ± 0.012 μm (mean value ± s.e.; *n* = 30).

### 3.2. RC-33/GG Interaction Product (IP)

GG was selected as the principal polymer of the composite system, especially due to its anionic nature. In the present work, the formation of a polyelectrolyte interaction product between GG and the cationic RC-33 has been investigated.

**Figure 3:**
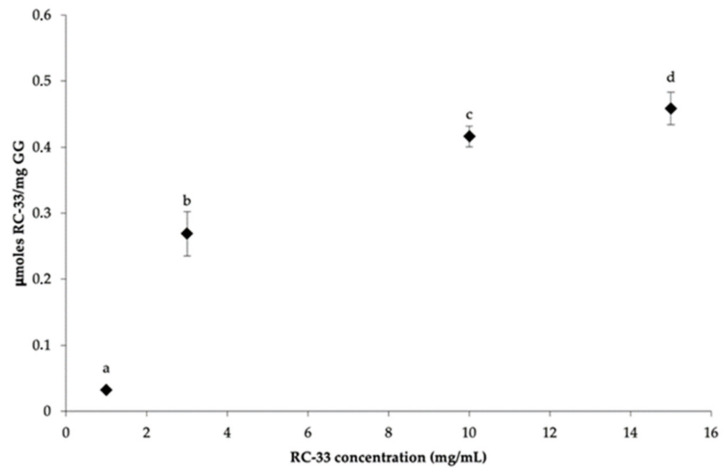
1-[3-(1,1′-biphen)-4-yl]butylpiperidine (RC-33) μmoles bond to 1 mg of gellan gum (GG) vs. RC-33 concentration profile (mean values ± s.d.; *n* = 3). One-way ANOVA, post hoc Scheffé test (*p* ≤ 0.05): a vs. b, c, d; b vs. c, d.

The results obtained from dialysis equilibrium studies are shown in Figure 3. In particular, RC-33 μmoles bond to 1 mg of GG are reported as a function of RC-33 concentration (mg/mL). GG concentration was fixed and equal to 1.5% *w*/*v*. For all the drug concentrations considered, a precipitate (mean particle diameter ± s.d.; *n* = 30: 42.51 ± 9.05 μm) forms in the dialysis bag, indicating the formation of an IP between RC-33 and GG. It can be observed that for RC-33 concentrations higher than 10 mg/mL, the drug amount bound to 1 mg of GG does not significantly change, indicating that the maximum binding capacity of GG is reached. This is equal to 0.416 mg of RC-33 for 1 mg of GG. 

**Figure 4:**
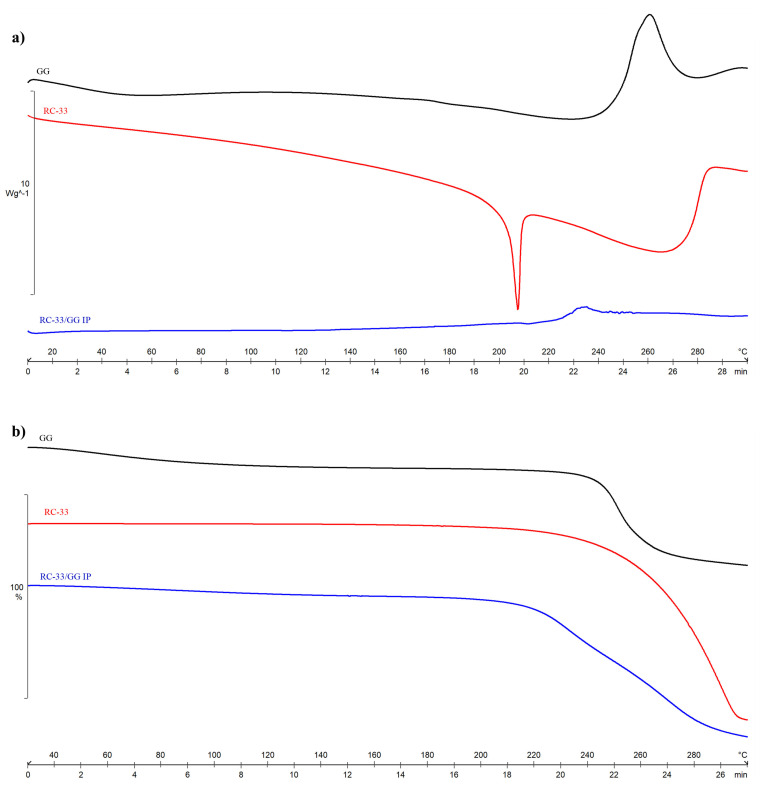
DSC (**a**) and TGA (**b**) curves of GG, RC-33 and RC-33/GG IP. Black line: GG, red line: RC-33 and blue line: RC-33/GG interaction product (IP).

In Figure 4, the DSC (a) and TGA (b) profiles of RC-33/GG IP are compared with those of pure GG and RC-33.

The DSC curve (a) of pure GG shows a broad endothermic peak at around 50 °C corresponding to the loss of water, as confirmed by the mass loss of about 8% recorded in the same temperature range in TGA curve (b). An exothermic effect at around 260 °C is also observed, indicating the decomposition of the polymer (T_onset, dec_ = 220.2 ± 0.3 °C in TGA curve).

As shown in Figure 4a, the thermal behavior of RC-33 is instead typical of an anhydrous crystalline sample that melts with an endothermic effect at 209.5 ± 0.8 °C (ΔH_melt_ = 135 ± 3 J·g^−1^), followed by sample degradation as confirmed by TGA curve (Figure 4b), where only a mass loss is recorded at about 207 °C, after the melting temperature.

In the DSC thermogram of RC-33/GG IP, the absence of the drug melting peak confirms that the drug is intimately mixed with GG to form IP. The endothermic and exothermic effects starting at about 220 °C can be attributed to the IP decomposition. This result is confirmed by TGA curve, where a mass loss in two steps is recorded in the same temperature range.

**Figure 5:**
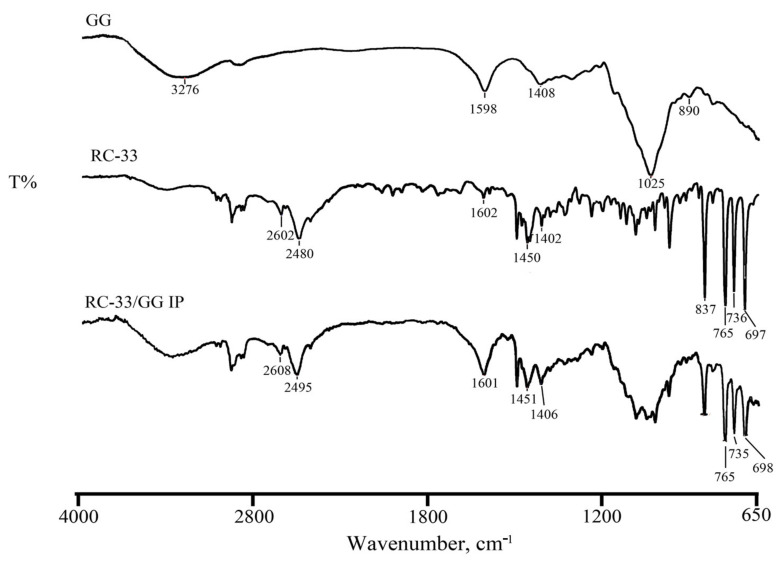
FT-IR spectra of GG, RC-33 and RC-33/GG IP.

In Figure 5, the FT-IR spectra of RC-33, GG and RC-33/GG IP are reported.

In FT-IR spectrum of pure GG, the bands at 1598 and 1408 cm^−1^ are due to asymmetric and symmetric stretching of carboxylate group. The band at 2896 cm^−1^ is due to the stretching vibrations of -CH_2_ group, while those at 1148 and 1024 cm^−1^ are due to ethereal and hydroxylic C-O stretching. Bending vibration of C-H appeared at 890 cm^−1^. The large band at around 3200–3400 cm^−1^ is due to the presence of OH- group of glucopyranose ring. As for the drug, the characteristic vibration bands appear at 2602 and 2480 cm^−1^, at 1602, 1450 and 1402 cm^−1^ in the region of C-C and C-N stretching in the ring, respectively. Moreover, three bands at 765, 736 and 697 cm^−1^ in the region of C-H bending are observed.

In the RC-33/GG IP spectrum, the shift of the RC-33 bands at 2602 and 2480 cm^−1^ to higher wavenumbers can be detected, confirming the thermal data.

### 3.3. Freeze-Dried Matrices (MXs)

**Figure 6:**
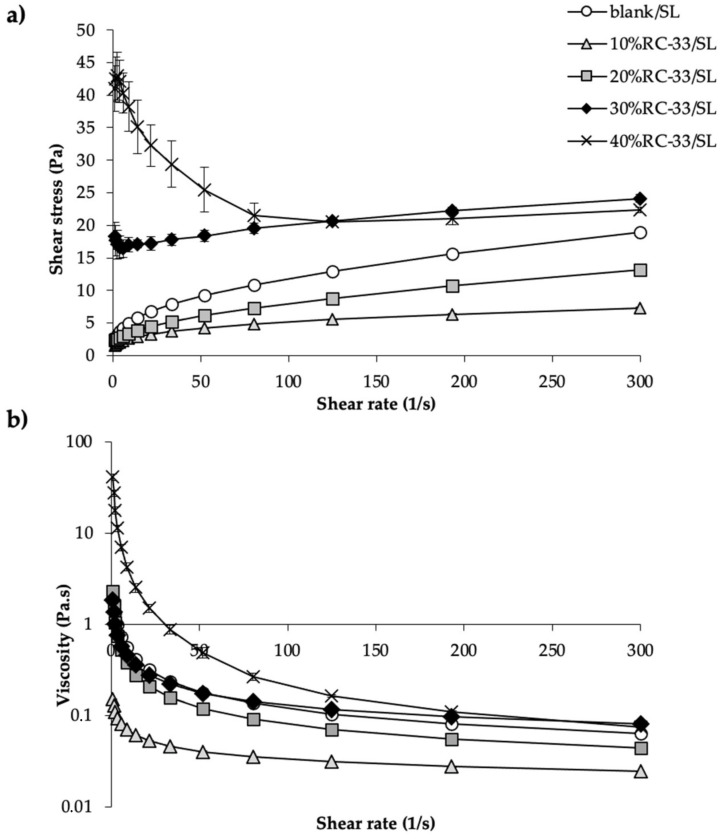
Flow (**a**) and viscosity profiles (**b**) of 1.5% *w*/*w* GG solution loaded with different amounts of RC-33 corresponding to 10, 20, 30 and 40% of the GG binding sites (respectively, 10%RC-33/solution (SL), 20%RC-33/SL, 30%RC-33/SL and 40%RC-33/SL); a 1.5% *w*/*w* GG solution free of RC-33 (blank/SL) was used as reference (mean values ± s.d.; *n* = 3). All the solution also contained glycine (Gly) at 0.5% *w*/*w*.

In Figure 6, flow and viscosity profiles of GG SLs containing increasing amount of RC-33, which correspond to 10%, 20%, 30% and 40% (10%RC-33/SL, 20%RC-33/SL, 30% RC-33/SL and 40%RC-33/SL) of GG binding sites according to the above cited GG maximum binding capacity, are compared with those of a GG solution free of RC-33 (blank/SL). The polymeric solutions also contained glycine at 0.5% *w*/*w*, a cryoprotectant agent [46,47,48], in view of their employment for matrix preparation by freeze-drying. It can be observed that the addition of a small amount of RC-33 to GG solution (10%RC-33/SL) is responsible for a decrease of viscosity at all the shear rates considered. This is attributable to the formation of an insoluble IP between GG and RC-33 that removes GG chains from the interaction with water. A further increase in RC-33 amount results in an increase in viscosity profiles that, in the case of 30%RC-33/SL and 40%RC-33/SL, are also higher than that observed for the solution free of RC-33 (blank/SL). In particular, the flow curves (Figure 6a) of 30%RC-33/SL and 40%RC-33/SL are characterized by an initial spur, more evident for the solution containing the highest RC-33 amount, meaning that the solution are highly structured as a result of an interaction of GG chains with IP.

Among all RC-33-loaded polymeric solutions considered, 20%RC-33/SL and 40% RC-33/SL, having, respectively, a lower and a higher viscosity with respect to GG solution free of RC-33, were selected for the continuation of the work and, thus, freeze-dried.

Freeze-drying is widely used for the fabrication of highly porous scaffolds intended for tissue regenerative purposes. Such a process is conducted in two stages. Firstly, polymeric solution is cooled down to a certain temperature. The formation of solvent crystals forces the polymeric chains to aggregate into the interstitial spaces. Once polymeric solution is in a frozen state, it is subjected to sublimation in order to allow solvent removal and the obtainment of an interconnected porous polymeric structure [7].

Then, 20%RC-33/MX and 40% RC-33/MX, endowed with porosity approximately equal to 80%, are characterized by a diameter of 2 cm and a thickness of 5 mm. The solid-state characterization performed on both RC-33/MXs confirms the presence of RC-33/GG IP into the matrices (see Supplementary Data: Figures S2 and S3).

**Figure 7:**
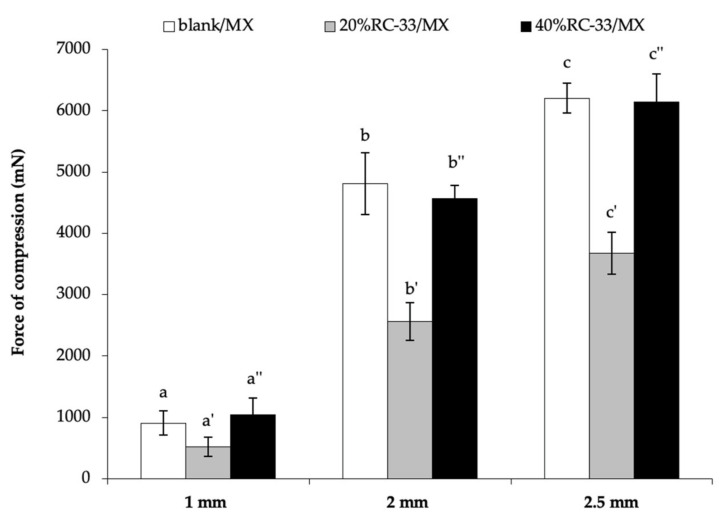
Compression force values of freeze-dried matrices loaded with RC-33 amounts corresponding to 20% and 40% of the GG interaction sites (respectively, 20% RC-33-loaded freeze-dried matrix (RC-33/MX) and 40% RC-33/MX); blank/freeze-dried matrix (MX) was used as reference (mean values ± s.e.; *n* = 3). One-way ANOVA; post hoc Scheffé test (*p* ≤ 0.05): b vs. b’; b’ vs. b”; c vs. c’; c’ vs. c”.

In Figure 7, the results obtained from compression measurements performed on RC-33/MXs are reported. Such measurements were intended to investigate MXs resistance to compressive forces to which MXs could be subjected throughout regular motion once applied at the site of injury. Blank/MX was used as reference. It can be observed that the force of compression, that is the force necessary for a probe to penetrate to a fixed depth inside the sample, decreases in presence of an amount of RC-33 corresponding to 20% of GG interaction sites (20%RC-33/MX). This is probably due to the formation of RC-33/GG IP. A further increase in RC-33 amount (40%RC-33/MX) produces an increase in such parameter. This behavior is in line with the results obtained from the rheological analysis performed on polymeric solutions containing different amounts of RC-33 (see Figure 6).

**Figure 8:**
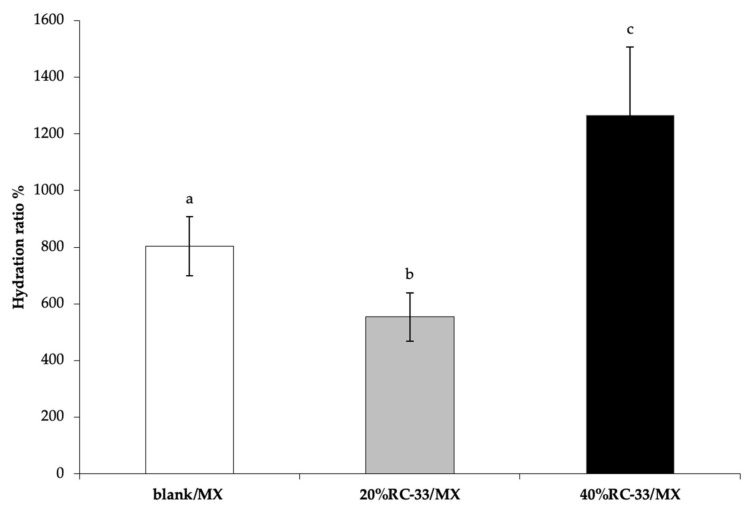
Hydration ratio % values of MXs loaded with RC-33 amounts corresponding to 20 and 40% of the GG interaction sites (respectively, 20%RC-33/MX and 40%RC-33/MX); blank/MX was used as reference (mean values ± s.e.; *n* = 3). One-way ANOVA; post hoc Scheffé test (*p* ≤ 0.05): b vs. c.

In Figure 8, the hydration ratio % values, calculated for the RC-33/MXs upon 6 h hydration, are compared with that obtained for the blank/MX. It can be observed that all the samples have the capability to absorb a high pH 7.4 PBS amount; after application at the site of injury, the highly porous structure of these MXs could allow the absorption and the drainage of biological fluids, with the consequent formation of a gel. It is widely reported in literature that gels represent attractive neural scaffolds, resembling the hydrated ECM microenvironment, especially in terms of physical and mechanical properties; therefore, gels allow the creation of an ideal architectural template in which cells can migrate [11].

However, a different hydration is observed depending on the amount of RC-33. For a small RC-33 amount (20%RC-33/MX) a decrease of hydration ratio % value is recorded: the GG chains involved in IP formation are not available for the hydration/swelling process. An increase in RC-33 amount (40%RC-33/MX) is responsible for enhanced hydration properties. These results also point out the occurrence of a different interaction between RC-33 and GG, depending on the RC-33 amount.

**Figure 9:**
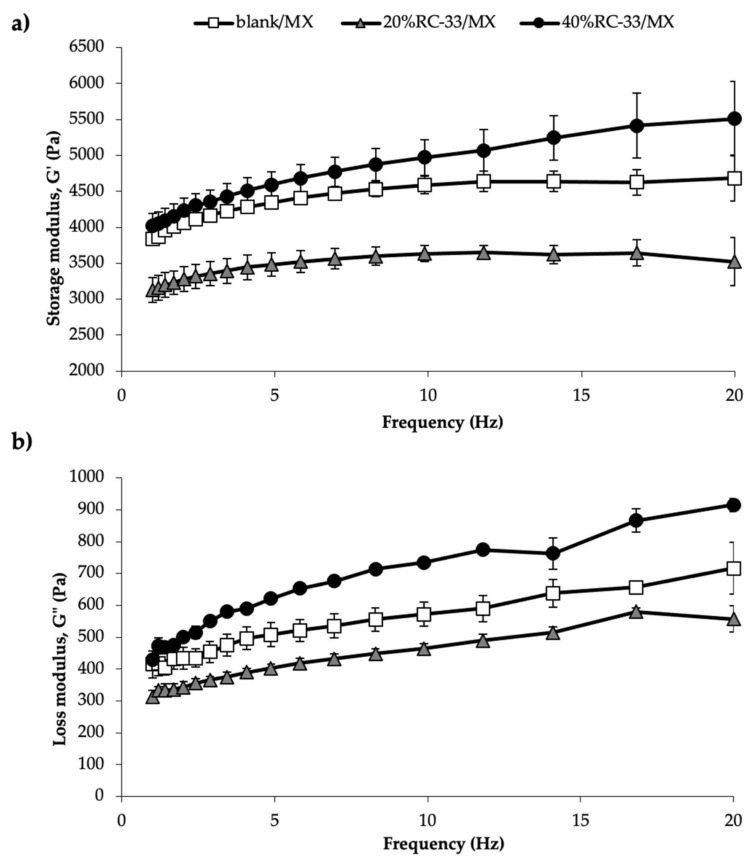
Storage (G’) (**a**) and loss (G’’) (**b**) modulus vs. frequency profiles of matrices free of RC-33 (blank/MX) and loaded with RC-33 amounts corresponding to 20% and 40% of the GG interaction sites (respectively, 20%RC-33/MX and 40%RC-33/MX) upon 6 h hydration in pH 7.4 PBS; blank/MX was used as reference (mean values ± s.e.; *n* = 3).

Figure 9 shows the viscoelastic properties of blank/MX, 20%RC-33/MX and 40%RC-33/MX upon 6 h hydration. It can be observed that all the samples show G’ values higher than G” ones in the whole range of frequencies considered. This indicates that all the samples are characterized by a prevalence of the elastic properties on the viscous ones. Analogously to what previously observed, the presence of different amounts of RC-33 produces different behaviors: A decrease of G’ and G’’ profiles is observed for a RC-33 amount corresponding to 20% of GG interaction sites (20%RC-33/MX); vice-versa, an increase in sample viscous and elastic properties occurs for a higher amount of RC-33. In particular, 40%RC-33/MX is characterized by an elastic modulus ranging from 4000 to 5500 Pa, depending on the frequency applied. Moreover, 40%RC-33/MX, when subjected to compression force, shows an E modulus equal to 428 ± 42 (mean value ± s.e.; *n* = 3). Such value is in the range proposed in the literature as optimal to promote neuronal cell differentiation [49,50].

**Figure 10:**
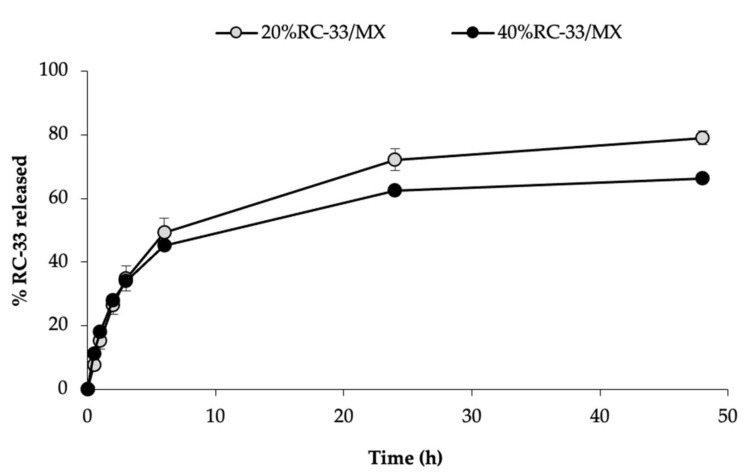
RC-33 release profiles of matrices loaded with RC-33 amounts corresponding to 20 and 40% of the GG interaction sites (respectively, 20%RC-33/MX and 40%RC-33/MX) (mean values ± s.e.; *n* = 9).

In Figure 10, % RC-33 released from 20%RC-33/MX and 40%RC-33/MX is reported vs. time. It can be observed that both the RC-33/MXs are able to prolong the release of RC-33. In particular, the matrix loaded with the highest amount of RC-33 (40%RC-33/MX) is characterized by a lower profile. This behavior is in line with the results previously obtained that pointed out that such a MX is more structured than that loaded with an amount of RC-33 corresponding to 20% of the GG sites (20%RC-33/MX).

Rossi and colleagues [35] have investigated the effect of RC-33 on neural growth factor (NGF)-induced neurite outgrowth in PC12 cell line. In vitro studies demonstrated that the drug candidate was able to increase the % of cells with neurite outgrowth (from 0.01 to 5 μM) and to enhance neurite elongation (from 0.25 to 5 μM).

According to our results, after 30 minutes, 20%RC-33/MX and 40%RC-33/M released an amount of RC-33 corresponding to 0.58 μM and 1.72 μM, respectively (*n* = 9). Such concentrations seem to be promising for further investigations on the efficacy of RC-33/MX on model cell lines.

### 3.4. RC-33/MX Containing CaCl_2_ (RC-33/MX + CaCl_2_)

In order to investigate if the incorporation of CaCl_2_ in 40%RC-33/MX could further slow-down RC-33 release due to the capability of calcium ions to cross-link GG chains, MX containing RC-33 corresponding to 40% of the GG interaction sites and CaCl_2_ (40%RC-33/MX + CaCl_2_) were prepared and characterized.

In Table 2, the results of the characterization of 40%RC-33/MX, prepared in presence and absence of CaCl_2_, are reported. It can be observed that the presence of CaCl_2_ significantly increases the mechanical resistance before hydration and decreases the hydration ratio %. As expected, due to the cross-link’s formation, 40%RC-33/MX + CaCl_2_ is characterized upon hydration by more pronounced viscoelastic properties.

**Figure 11:**
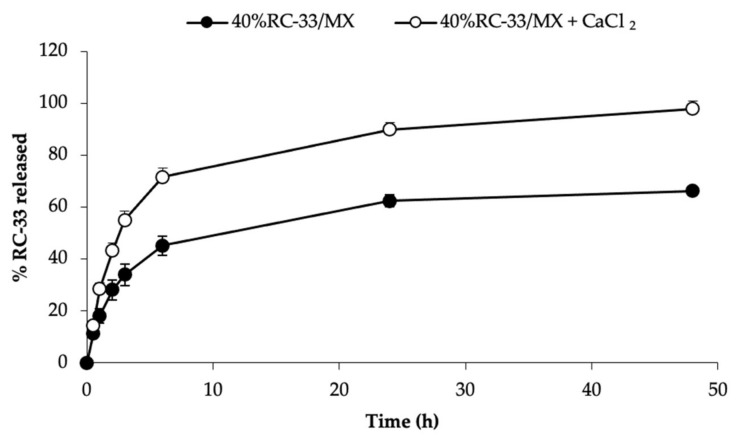
RC-33 release profiles of freeze-dried matrices loaded with RC-33 amounts corresponding to 40% of the GG interaction sites in absence (40%RC-33/MX) and in presence of CaCl_2_ (40%RC-33/MX + CaCl_2_) (mean values ± s.e.; *n* = 9).

In Figure 11, RC-33 release profiles of 40%RC-33/MX, in presence (40%RC-33/MX + CaCl_2_) and in absence (40%RC-33/MX) of CaCl_2_, are compared. Although the presence of calcium ions is responsible for the formation of a hydrated MX characterized by increased viscoelastic properties, the RC-33 release from 40%RC-33/MX + CaCl_2_ is speeded up. These results could be due to the displacement action of calcium ions towards RC-33 linked to GG chains.

Such hypothesis was confirmed by the results of dialysis equilibrium studies performed on 40%RC-33/SL by using saline with and without calcium ions as outer medium (see Supplementary Data).

### 3.5. Composite System (cNFs + 40%RC-33/MX)

**Figure 12:**
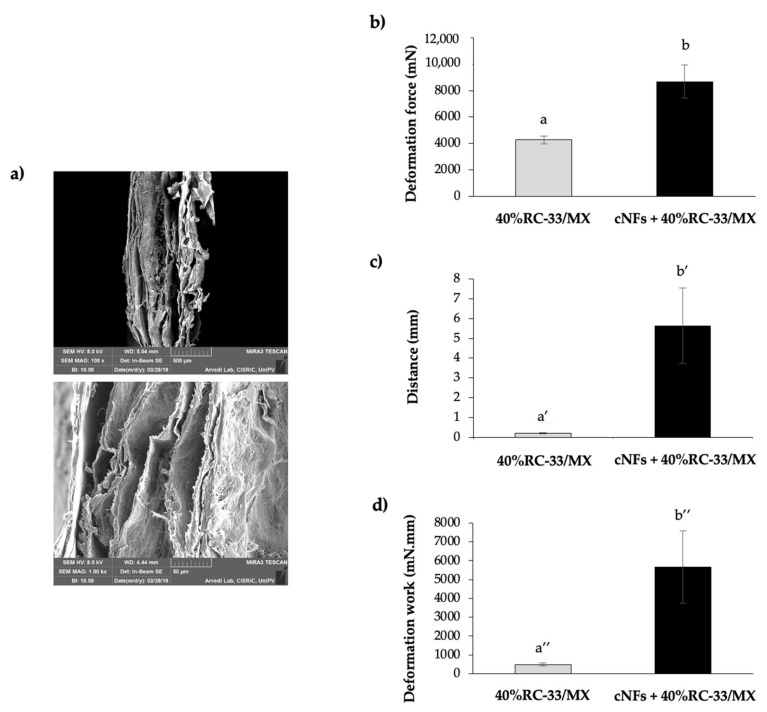
SEM micrographs at two different magnifications (100X at the top and 1.00 kX at the bottom) of composite system cNFs + 40%RC-33/MX (**a**); deformation force (**b**), distance (**c**) and deformation work (**d**); 40%RC-33/MX, in absence of cNFs, was used as reference (mean values ± s.e.; *n* = 3). Different symbols denote statistically different data (*p* ≤ 0.05; One-way ANOVA one-way; *t*-test).

Finally, cNFs + 40%RC-33/MX was prepared. After cross-linking, cNFs were soaked in a GG-based solution, containing an RC-33 amount corresponding to 40% of GG binding sites (40%RC-33/SL), and then subjected to freeze-drying process. In Figure 12a, SEM micrographs show how the composite system is configured; in particular, in the image at the bottom it can be appreciated the inner structure of the system.

The results obtained from the tensile test performed on cNFs + 40%RC-33/MX are reported in Figure 12b–d. It can be observed that the presence of cNFs is responsible for an increase in all the three relevant parameters: deformation force, distance and deformation work. These results indicate that the presence of the cNFs is responsible for a marked improvement of the MX mechanical resistance. The increase in distance in presence of cNFs points out that also the system elasticity is enhanced. Such results were also confirmed by the work of Liu and colleagues, who developed a system composed of chitosan fibers incorporated into an acetylated GG-based hydrogel. System mechanical properties, evaluated by oscillatory rheological measurements as a function of frequency, increase with the increasing content of chitosan fibers; the storage moduli (G’; at 1 rad/s), reflecting the elasticity of the system, is approximately 4.6 times more than that of the hydrogel without fibers [51].

## 4. Conclusions

In the present work, a composite system for the local delivery of the neuroprotective S1R agonist, RC-33, was developed as a potential tool for the treatment of nervous tissue injuries. The system, consisting of cross-linked electrospun nanofibers embedded in a RC-33-loaded freeze-dried matrix, was designed to bridge the lesion gap, control drug delivery and enhance axonal regrowth. Such a system was easy to handle and shapeable according to the lesion size and morphology.

cNFs represent the inner structure of the composite system. They were prepared through the electrospinning technique starting from polymeric solutions, in which GG was blended with a mixture of two different grades of PEO and poloxamer. Due to their best electrospinnability and highest size homogeneity, G4 nanofibers were selected as the most promising formulation and, thus, cross-linked in calcium ions to guarantee their temporary insolubility in aqueous media (cNFs).

MXs loaded with different amounts of RC-33 and obtained through a freeze-drying process, provide the outer structure of the composite system. The formation of an interaction product between GG and RC-33 was confirmed by both dialysis equilibrium studies and solid-state investigations. Depending on drug candidate concentration, IP differently affected MX mechanical properties. Furthermore, 40%RC-33/MX, containing an amount of RC-33 corresponding to 40% of the GG interaction sites, showed a good resistance to penetration, probably due to the high structuring of the starting polymeric solution.

After hydration, RC-33/MXs were characterized by the capability to absorb and retain a high buffer content, forming a gel with marked viscoelastic properties that well resembled the native hydrated ECM. In particular, 40%RC-33/MX upon hydration was characterized by an elasticity recognized as optimal to promote neuronal cell differentiation. Once applied at the site of injury, the porous structure of the system should allow the absorption and the drainage of biological fluids; the resulting hydrated system was able to release the drug candidate in a controlled manner.

Moreover, 40%RC-33/MX was also prepared in presence of CaCl_2_ in order to further increase both the mechanical and hydration properties of the system. Nevertheless, the RC-33 release from MX containing CaCl_2_ is speeded up, probably due to the displacement action of calcium ions towards RC-33 linked to GG chains.

Finally, a composite scaffold was successfully prepared: the presence of cross-linked nanofibers increased system mechanical resistance. As a future perspective, the performance of the composite system will be evaluated through studies on model cell lines.

## Figures and Tables

**Table 1:** Composition of the polymeric solutions for electrospinning, expressed as % *w*/*w*.

Formulations	GG	l-PEO	h-PEO	P407
G1	1.5	1.3	0.133	2
G2	1.5	1.5	0.056	2
G3	1.5	1.98	0.15	2
G4	1.5	2.2	0.15	2

**Table 2:** Results of the characterization of 40%RC-33/MX, in presence and absence of CaCl_2_ (mean values ± s.e.; *n* = 3). *t*-test: a vs. a’; b vs. b’; c vs. c’; d vs. d’.

40%RC-33/MX	Matrix Properties			
	Deformation Force (mN) at 1 mm Depth	Hydration Ratio %	G’ (Pa) at 10 Hz after Hydration	G” (Pa) at 10 Hz after Hydration
In presence of CaCl_2_	*1655 ^a^* (± 263)	*530 ^b^* (± 23)	*8920 ^c^* (± 2117)	*1062 ^d^* (± 213)
In absence of CaCl_2_	*1043 ^a’^* (± 272)	*1265*^*b*’^ (± 242)	*4973 ^c’^* (± 248)	*735 ^d’^* (± 5)

## Data Availability

Data is contained within the supplementary material.

## Supplementary Materials

The following are available online at https://www.mdpi.com/1999-4923/13/2/164/s1, Figure S1: Rheological properties of G1–G4 polymeric solutions intended to be electrospun: (a) viscosity values measured at 1000 s^−1^; (b) storage modulus (G’) and loss modulus (G”) values measured at 10 Hz.; (c) loss tangent (tg delta) values measured at 10 Hz (mean values ± s.d.; *n* = 3). The same trend of viscoelastic parameters has been observed in the range 0.1–10 Hz; Figure S2: DSC (a) and TGA (b) curves of Gly, blank/MX, 20%RC-33/MX and 40%RC-33/MX. Black line: Gly; red line: Blank/MX; blue line: 20%RC-33/MX; green line: 40%RC-33/MX; Figure S3: FT-IR spectra of Gly, blank/MX, 20%RC-33/MX and 40%RC-33/MX. Black line: Gly; red line: blank/MX; blue line: 20%RC-33/MX; green line: 40%RC-33/MX; Figure S4: RC-33 amounts dissolved in the outer medium, in presence (saline + CaCl_2_) or in absence of CaCl_2_ (saline), on time (mean values ± s.e.; *n* = 3).
