# Supplementary material for: Gellan-Based Composite System as a Potential Tool for the Treatment of Nervous Tissue Injuries: Cross-Linked Electrospun Nanofibers Embedded in a RC-33-Loaded Freeze-Dried Matrix
Source: Pharmaceutics. 2021 Jan 26;13(2):164. doi: 10.3390/pharmaceutics13020164 (PMC7912322; doi:10.3390/pharmaceutics13020164)
Supplement: Supplementary file 1 [file pharmaceutics-13-00164-s001.pdf]

pharmaceutics

Supplementary Materials: Gellan-based Composite System as a Potential Tool for the Treatment of Nervous Tissue Injuries: Cross-linked Electrospun Nanofibers Embedded in a RC-33-Loaded Freeze-dried Matrix

Barbara Vigani, Caterina Valentino, Valeria Cavalloro, Laura Catenacci, Milena Sorrenti, Giuseppina Sandri, Maria Cristina Bonferoni, Chiara Bozzi, Simona Collina, Silvia Rossi * and Franca Ferrari

Citation: Electrospinning, C.o.t.P.S.f. Barbara Vigani, Caterina Valentino, Valeria Cavalloro, Laura Catenacci, Milena Sorrenti, Giuseppina Sandri, Maria Cristina Bonferoni, Chiara Bozzi, Simona Collina, Silvia Rossi * and Franca Ferrari. *Pharmaceutics* **2021**, *13*, 164. https://doi.org/10.3390/pharmaceutics13020164

Received: 18 December 2020
Accepted: 21 January 2021
Published: 26 January 2021

Publisher's Note: MDPI stays neutral with regard to jurisdictional claims in published maps and institutional affiliations.

Copyright: © 2021 by the author. Licensee MDPI, Basel, Switzerland. This article is an open access article distributed under the terms and conditions of the Creative Commons Attribution (CC BY) license (http://creativecommons.org/licenses/by/4.0/).

Characterization of the Polymeric Solutions for Electrospinning

1. Methods

All the polymeric solutions (G1–G4) were characterized in terms of rheological properties (viscosity and viscoelastic measurements) and conductivity, as reported in [1,2].

1.1. Rheological Measurements

Rheological analyses were carried out by means of a rotational rheometer (MCR 102, Anton Paar, Turin, Italy) equipped with a cone plate combination (CP50-1, diameter = 50 mm; angle = 1°) as measuring system.

Sample viscosity was investigated at increasing shear rates in the range 1–1000 s^{-1} at 33°; three replicates were affected for each polymeric solution.

Sample viscoelasticity was, instead, evaluated through dynamic oscillatory measurements at 33 °C: a stress sweep test and an oscillation test were carried out. In the stress sweep test, increasing stresses were applied at a constant frequency (1 Hz) and the elastic response of the sample, expressed as storage modulus G′, was measured. In the oscillation test, a shear stress, chosen in the linear viscoelastic region previously determined in the stress sweep test, was applied at increasing frequencies (1 to 20 Hz) and G′ (storage modulus) and G″ (loss modulus) profiles were recorded. Loss tangent value (tgδ) was calculated as G″/G′ at 10 Hz. Three replicates were considered for each polymeric solution.

1.2. Conductivity Measurements

Sample conductivity was measured with Mettler ToledoTM FiveGoTM F3 conductivity meter apparatus (Fisher Scientific, Milan, Italy). Three replicates were considered for each solution.

2. Results and Discussion

2.1. Characterization of the Polymeric Solutions for Electrospinning

In Figure S1, the results of the rheological analyses performed on G1–G4 polymeric solutions are reported. The evaluation of polymeric solution viscoelasticity is legitimate since poly (ethylene oxide) (PEO) is characterized by well-known viscoelastic properties.

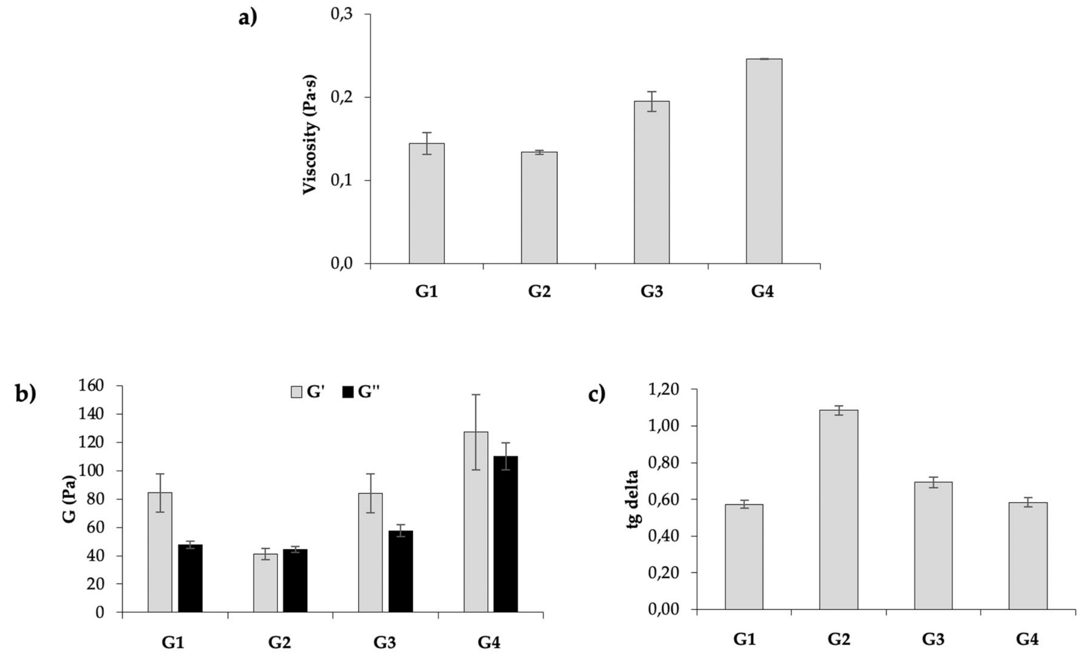

Figure S1. Rheological properties of G1–G4 polymeric solutions intended to be electrospun: (**a**) viscosity values measured at 1000 s^{-1}; (**b**) storage modulus (G′) and loss modulus (G″) values measured at 10 Hz.; (**c**) loss tangent (tg delta) values measured at 10 Hz (mean values ± s.d.; *n* = 3). The same trend of viscoelastic parameters has been observed in the range 0.1–10 Hz.

As expected, the viscosity value increases on increasing total PEO concentration (Figure S1a). Analogously, also the loss modulus G″, expression of the viscous contribution to sample viscoelastic properties, depends on total PEO concentration. The trend is similar to that observed for the viscosity values. As for the elastic properties, indicated by the storage modulus (G′), they are strongly dependent on the concentration of high molecular weight (MW) PEO (h-PEO): the lowest G′ value is observed for G2 solution characterized by the lowest h-PEO concentration (Figure S1b). Moreover, G2 is also the solution characterized by the highest loss tangent value (higher than 1) (Figure S1c). Since such parameter is calculated as the ratio between G″ and G′, a tg delta value higher than one means that the viscous component prevails on the elastic one: the lower loss tangent value, the higher is the importance of the elastic behavior on the viscous one.

Moreover, all the solutions are characterized by conductivity values ranging into the interval 815–850 μS/cm, lower than the value measured for a 1.5% w/w pure gellan gum (GG) solution that is equal to 980 ± 2 μS/cm (*n* = 3; ± s.e.). This behavior is probably due to a shielding effect of PEO macromolecules on the charged GG chains [1, 2].

Solid-state Characterization of Freeze-dried Matrices (MXs)

1. Methods

Differential Scanning Calorimetry (DSC), thermogravimetric (TGA) and Fourier-transform infrared spectroscopy (FT-IR) analyses were performed on blank/ freeze-dried matrix (MX) and 1-[3-(1,1′-biphen)-4-yl]butylpiperidine (RC-33)-loaded freeze-dried matrices (RC-33/MXs) as described in the Section 2.3.2.

2. Results and Discussion

In Figure S2, the DSC (a) and TGA (b) profiles of 20%RC-33/MX and 40%RC-33/MX are compared with those of blank/MX and pure glycine (Gly).

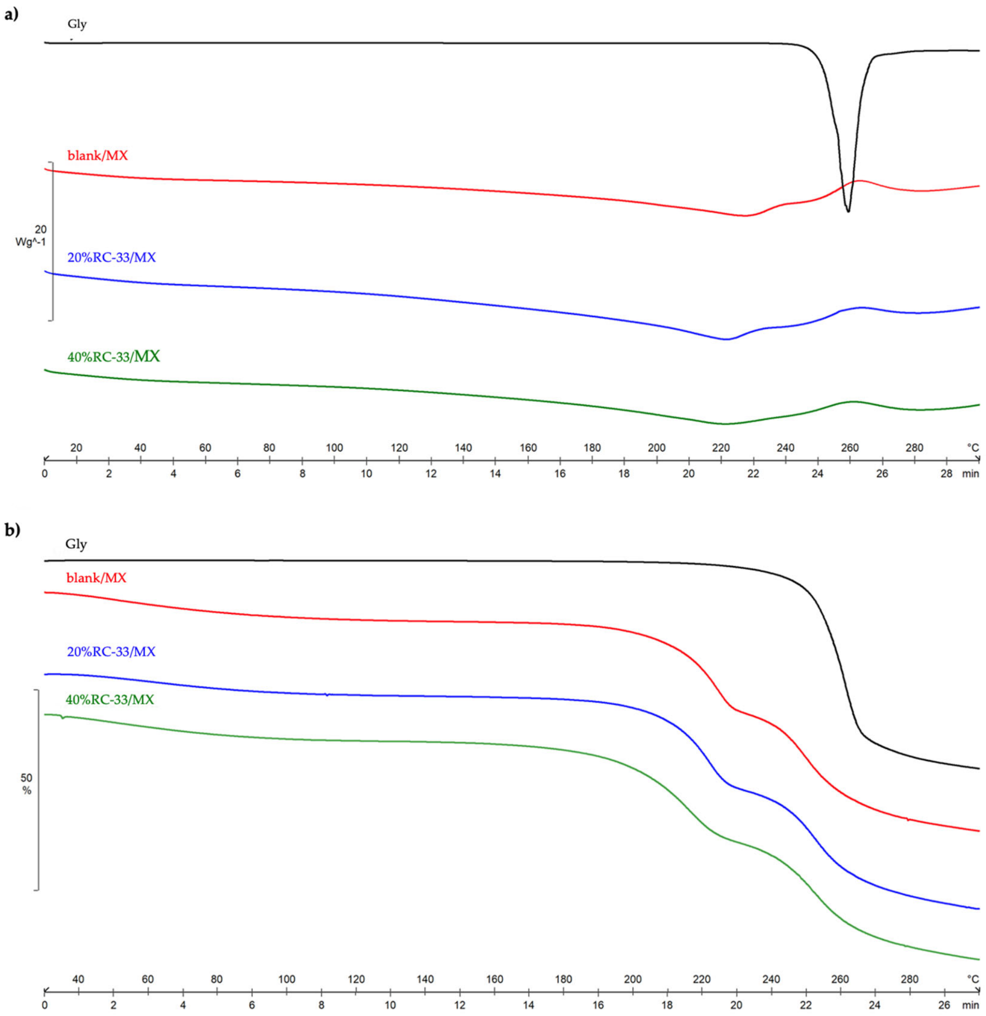

Figure S2. DSC (**a**) and TGA (**b**) curves of glycine (Gly), blank/ freeze-dried matrix (MX), 20%1-[3-(1,1′-biphen)-4-yl]butylpiperidine (RC-33)-loaded freeze-dried matrix (RC-33/MX) and 40%RC-33/MX. Black line: Gly; red line: blank/MX; blue line: 20%RC-33/MX; green line: 40%RC-33/MX.

The Gly shows a sharp endotherm peak at 252.2 ± 0.8 °C due to melting concomitant with decomposition of the sample, as revealed by the TGA mass loss ((Tonset, dec = 207.9 ± 0.4 °C).

The DSC thermograms of the blank/MX and RC-33/MXs show a profile typical of amorphous sample, where broad endothermic effects due to dehydration, at lower temperature (mass loss of about 7% by TGA), and sample degradation, at higher temperature, are evident. The absence of drug melting peak in DSC thermograms of 20%RC-33/MX and 40%RC-33/MX confirms that the interaction product (IP) is dispersed in the MXs.

The TGA curve of MX with higher drug loading (40%RC-33/MX) shows the lowest thermal stability as confirmed by the lower onset temperature of decomposition.

In FT-IR spectrum of Gly (Figure S3), the bands due to the -COO group are observed at 1597 and 1411 cm^{-1}; the band at 1502 cm^{-1} may be assigned to the NH group bending, while the bands at 1443 and 1332 cm^{-1} to CH_2 bending and twisting.

FTIR spectrum of the blank/MX is observed to be a combination of the spectra of GG (see Figure S4) and Gly. The presence of IP in the RC-33/MXs is instead confirmed by the appearing in their spectra of some characteristic vibration bands of the drug, in particular those at 765, 736 and 697 cm^{-1}.

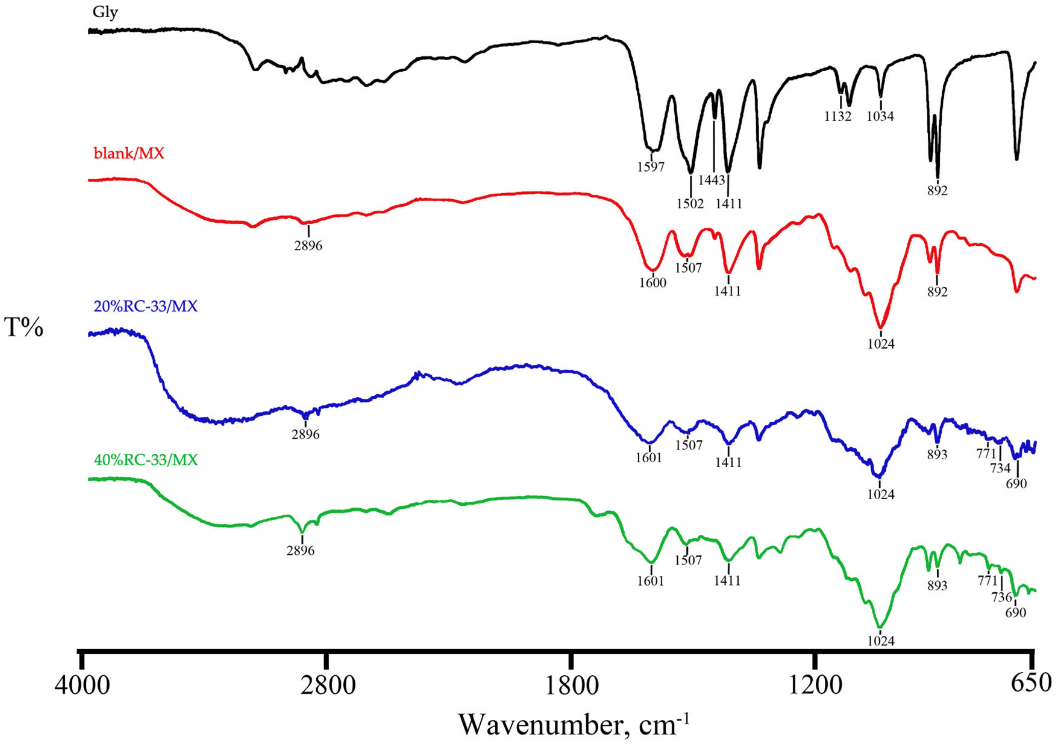

Figure S3. FT-IR spectra of Gly, blank/MX, 20%RC-33/MX and 40%RC-33/MX. Black line: Gly; red line: blank/MX; blue line: 20%RC-33/MX; green line: 40%RC-33/MX.

RC-33/GG Binding Study by Dialysis Equilibrium in Presence of $CaCl_2$

1. Methods

Dialysis equilibrium studies were performed to investigate the displacement action of calcium ions towards RC-33 linked to GG chains. Briefly, a solution, containing 1.5% w/w GG, 0.5% w/w Gly and 0.25% w/w RC-33 (40%S), was enclosed in a dialysis bag (seamless cellulose tubing, cut-off > 12,000 Da; Sigma Aldrich, Milan, Italy) and, then, dialyzed towards saline with or without $CaCl_2$ at the concentration of 0.035% w/w (calculated so that Ca^{2+} could occupy 30% of the remaining GG binding sites) under mild stirring at room temperature until 24 h.

The dialysis bag did not allow the polymer to get out, but allowed the drug to diffuse out and the calcium ions to diffuse in. Drug concentration outside the dialysis bags was quantified by a HPLC method as described in the Section 2.3.1.

2. Rsults and Discussion

In Figure S4, RC-33 amounts (mg) dissolved in the outer medium on time are reported. It can be observed that the presence of $CaCl_2$ in the outer medium is responsible for higher free RC-33 amounts. These results confirm the capability of calcium ions to remove drug from the IP with GG.

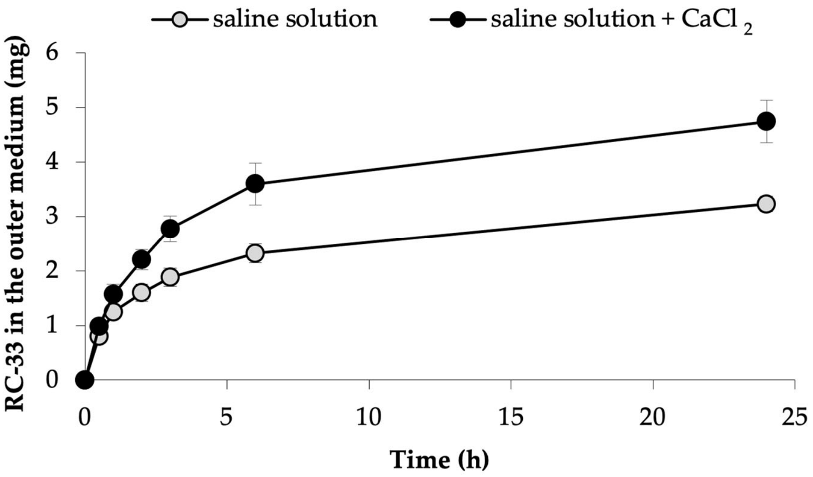

Figure S4. RC-33 amounts dissolved in the outer medium, in presence (saline + $CaCl_2$) or in absence of $CaCl_2$ (saline), on time (mean values ± s.e.; $n = 3$).

References

1. Vigani, B.; Rossi, S.; Sandri, G.; Bonferoni, M.C.; Milanesi, G.; Bruni, G.; Ferrari, F. Coated electrospun alginate-containing fibers as novel delivery systems for regenerative purposes. *Int. J. Nanomed.* **2018**, *13*, 6531–6550, doi:10.2147/ijn.s175069.
2. Vigani, B.; Rossi, S.; Milanesi, G.; Bonferoni, M.C.; Sandri, G.; Bruni, G.; Ferrari, F. Electrospun Alginate Fibers: Mixing of Two Different Poly(ethylene oxide) Grades to Improve Fiber Functional Properties. *Nanomaterials* **2018**, *8*, 971, doi:10.3390/nano8120971.
